# Ethanol exposure and high-fat diet: assessing the neuroimmune and metabolic mechanisms in Alzheimer’s disease pathology

**DOI:** 10.3389/fnins.2026.1837438

**Published:** 2026-06-03

**Authors:** George Chigozie Njoku, Viswanathan Saraswathi

**Affiliations:** 1 Department of Internal Medicine, Division of Diabetes, Endocrinology, and Metabolism, University of Nebraska Medical Center, Omaha, NE, United States; 2 VA Nebraska-Western Iowa Health Care System, Omaha, NE, United States

**Keywords:** Alzheimer’s disease, Aβ clearance, ethanol, high-fat diet, neuroinflammation, Tau

## Abstract

Alzheimer’s disease (AD) is a chronic, progressive neurodegenerative disorder characterized by the accumulation of amyloid-β (Aβ) plaques and hyperphosphorylated Tau, leading to neurofibrillary tangle formation and synaptic dysfunction. Increasing evidence indicates that these hallmark pathologies are shaped by sustained alterations in neuroimmune and metabolic homeostasis. Lifestyle-associated exposures, including ethanol consumption and high-fat diet (HFD) intake, are emerging as modulators of neuroinflammatory tone and may influence susceptibility to AD-like pathology across the lifespan. Preclinical studies show that ethanol exposure increases Aβ42/40 ratios, promotes oxidative stress, and activates Tau-associated kinases, whereas HFD induces insulin resistance, alters microglial lipid handling, and impairs Aβ clearance. These effects converge on overlapping pathways involving microglial activation, metabolic dysregulation, and kinase–phosphatase imbalance. Evidence directly examining combined ethanol and HFD exposure remains limited; however, available studies suggest co-occurring or additive effects on cognitive impairment, neuroinflammation, and synaptic dysfunction. This review synthesizes current evidence to delineate shared neuroimmune and metabolic mechanisms linking ethanol and HFD exposure to AD-related pathology. We highlight points of mechanistic overlap, including oxidative stress, inflammatory signaling, and disrupted proteostasis, while distinguishing between directly measured effects and inferred pathways. Finally, we identify key gaps in understanding how dual exposures interact and outline priorities for future studies aimed at clarifying their contribution to AD risk and progression.

## 1 Introduction

Alzheimer’s disease (AD) is characterized by progressive cognitive decline and is defined pathologically by the accumulation of amyloid-β (Aβ) and hyperphosphorylated Tau, which forms neurofibrillary tangles (NFTs) (Zhang et al., 2024; Zheng and Wang, 2025). Although these features remain central to the disorder, accumulating experimental and genetic evidence suggests that their development is shaped by sustained alterations in the brain’s immune environment (Chen and Yu, 2023; Zhang et al., 2024). Within this immune context, microglia and astrocytes respond to aggregated proteins through innate immune pathways, including phagocytic and pattern-recognition receptor (PRR) signaling, leading to persistent inflammatory activation that alters neuronal vulnerability and protein clearance (Han et al., 2025). In this framework, neuroinflammation is increasingly recognized as a key pathological process linking protein aggregation to neurodegeneration (Zhang et al., 2023). Chronic glial activation contributes to cytokine production, oxidative stress, mitochondrial dysfunction, and impaired proteostasis (Michalak and Michalak, 2025). These observations suggest that immune signaling may play an active role in shaping disease progression rather than functioning solely as a downstream response to neuronal injury (Han et al., 2025).

Lifestyle-related exposures that disrupt metabolic and inflammatory balance over time may engage these same neuroimmune pathways and influence AD risk across the lifespan (Livingston et al., 2024; Zheng and Wang, 2025). Ethanol consumption is one such modifiable exposure and has long been associated with cognitive impairment, neurodegeneration, and brain structural abnormalities across clinical and experimental studies. This association is supported by evidence from neuroimaging, postmortem analyses, and experimental models; evidence for region-specific neuronal injury is reported, though the extent of consistent neuronal loss in humans appears variable across studies (Chang et al., 2025; Topiwala et al., 2025). Mechanistically, ethanol promotes glial activation through toll-like receptor (TLR) signaling, increases proinflammatory cytokine production, and enhances oxidative stress (Warden et al., 2019). It also indirectly augments central inflammation via systemic immune activation and peripheral immune cell infiltration (Chang et al., 2025). These effects suggest that ethanol contributes to neuroimmune dysregulation in ways that may influence neuronal vulnerability and protein homeostasis, rather than acting solely as a secondary consequence of injury (García-Domínguez, 2025). In this context, ethanol engages neuroimmune pathways that overlap with those implicated in AD-like pathology, indicating that its effects extend beyond isolated toxicity and may influence broader disease-relevant mechanisms (Gołaszewski et al., 2025).

In Western societies, ethanol consumption frequently co-occurs with high-fat diet (HFD) intake, an exposure pattern with established neurocognitive consequences. Long-term HFD consumption promotes systemic inflammation and disrupted insulin signaling (Abbasi and Khodadadi, 2025; Hoffman et al., 2021) and exerts pronounced effects within brain regions supporting learning and memory (Gomes et al., 2025; Wang et al., 2020). The hippocampus, which relies on activity-dependent synaptic plasticity and remodeling, is among the earliest regions affected in AD and exhibits heightened basal immune activity and susceptibility to inflammatory insults relative to other brain areas (Gomes et al., 2025; Rao et al., 2022; Wang et al., 2020). Experimental studies demonstrate that even brief HFD exposure, as short as 3 days, is sufficient to induce hippocampal neuroinflammation; elevate proinflammatory cytokines such as interleukin-1β (IL-1β), IL-6, and tumor necrosis factor (TNF-α), impair long-term potentiation at Schaffer collateral-CA1 synapses; and produce measurable cognitive deficits in aged rodents (Spencer et al., 2017). These effects are closely linked to activation of nuclear factor kappa-light-chain-enhancer of activated B cells (NF-κB) signaling, and inhibition of its activation has been reported to confer protective effects in metabolic and neurological disease contexts (Cortez et al., 2013). HFD-induced neuroinflammation and central insulin resistance have also been proposed to impair Aβ clearance and accelerate Aβ deposition in AD mouse models (Wakabayashi et al., 2019).

Although ethanol exposure and HFD have each been linked to AD-related changes, the two areas of research have largely developed independently, with limited mechanistic integration. This raises the question of whether their effects are additive or whether they converge on shared pathways that increase neurodegenerative risk. In this review, we examine whether ethanol and HFD may act as convergent modifiers of AD-like pathology by influencing microglial activity, metabolic-lysosomal function, and kinase-phosphatase balance involved in Aβ and Tau regulation. We draw primarily on evidence from single-exposure studies and the limited available combined-exposure data to assess whether these mechanisms represent points of overlap. Although both exposures have been linked to neuroinflammation and alterations in Aβ and Tau homeostasis, direct evidence for their joint effects on these outcomes remains scarce. This framework is intended to organize existing findings and highlight areas requiring further experimental validation, particularly in models that directly assess proteinopathy under combined exposure conditions.

## 2 Ethanol exposure and Alzheimer-like pathology

### 2.1 Ethanol metabolism and systemic-brain interactions

Ethanol exposure perturbs central nervous system (CNS) function through a combination of systemic metabolic disruption and neuroimmune activation. Following ingestion, ethanol is primarily metabolized in the liver via alcohol dehydrogenase (ADH) and cytochrome P450 2E1 (CYP2E1) pathways, generating acetaldehyde and reactive oxygen species (ROS) that contribute to systemic oxidative stress (Jiang et al., 2020). Chronic or binge exposure promotes a pro-inflammatory peripheral environment, in part through disruption of gut barrier integrity, leading to increased intestinal permeability and endotoxemia, including translocation of lipopolysaccharide (LPS) into circulation (Antón et al., 2018; Chancharoenthana et al., 2025). These peripheral inflammatory signals can interface with the brain through the neurovascular unit, where ethanol-associated blood-brain barrier (BBB) dysfunction and neurovascular injury increase permeability to circulating mediators and facilitate leukocyte interactions (Carrino et al., 2021; Rodríguez-González et al., 2023; Togre et al., 2024; Vore and Deak, 2022). Even modest BBB disruption may amplify central innate immune signaling, particularly under conditions of sustained systemic inflammation (Carrino et al., 2021; Rodríguez-González et al., 2023; Togre et al., 2024; Vore and Deak, 2022).

### 2.2 Human and clinical evidence for AD markers in individuals with alcohol use disorder

Direct human evidence that ethanol exposure accelerates Aβ or Tau pathology remains limited, as most epidemiological and neuroimaging studies of ethanol consumption have focused on dementia incidence, brain atrophy, and white-matter integrity and only infrequently incorporate AD-defining biomarkers such as Aβ positron emission tomography, Tau imaging, or longitudinal cerebrospinal fluid measures stratified by ethanol exposure (Daviet et al., 2022; Topiwala et al., 2022a,b). Observational studies assessing circulating or cerebrospinal fluid Aβ in relation to ethanol intake report heterogeneous findings that vary by drinking pattern, age, and cohort characteristics, without consistent evidence of increased cerebral Aβ deposition or accelerated accumulation over time (Drouka et al., 2025). Consistent with this variability, population-based studies examining ethanol consumption and AD risk report mixed associations, ranging from increased risk and faster cognitive decline to null findings, and, in some cohorts, an apparent protective association, particularly with low-to-moderate intake (García et al., 2010; Harwood et al., 2010; Heymann et al., 2016; Luchsinger et al., 2004; Piazza-Gardner et al., 2013; Venkataraman et al., 2017; Weyerer et al., 2011). At present, a robust association between ethanol consumption and AD-related pathology is largely supported at the preclinical level rather than through direct human biomarker evidence.

### 2.3 Preclinical evidence from animal models

Preclinical studies indicate that ethanol exposure modifies AD-like pathology in a manner that depends on exposure pattern, developmental timing, and model background (Table 1). Across paradigms, effects are most consistently observed in microglial/immune activation and metabolic–lysosomal processes, with more variable but functionally relevant changes in intracellular signaling pathways linked to kinase–phosphatase balance. In 3xTg-AD mice, voluntary ethanol consumption is associated with region-selective increases in Aβ42/40 in the lateral entorhinal and prefrontal cortex, increased total Tau across multiple regions, and elevated p-Tau (Ser199/Ser202) in the hippocampus, together with reduced Akt/protein kinase B–mechanistic target of rapamycin (Akt/PKB–mTOR)–associated phosphoprotein signaling (Hoffman et al., 2019). Notably, these outcomes occur without consistent changes in APP processing, suggesting that ethanol primarily perturbs protein handling and turnover rather than uniformly increasing Aβ production. This pattern indicates that ethanol-related changes in Aβ and Tau are more likely driven by disrupted proteostatic regulation than by increased peptide generation.

**TABLE 1:** Preclinical studies examining ethanol exposure and AD–related pathology.

Model	Ethanol paradigm	Exposure window	Aβ /plaques	Tau endpoints	Neuroinflammation/ glia	Cognitive/ behavioral outcomes	Mechanistic notes (nodes)	References
3xTg-AD	Voluntary (2-bottle choice)	Adult	↑ Aβ42/40 (region-specific)	↑ Total Tau; ↑ p-Tau (Ser199/202)	↓ Akt/mTOR signaling	Behavioral deficits	Proteostasis/signaling disruption	Hoffman et al., 2019
3xTg-AD	AIE (5 g/kg, P25–55)	Adolescent → adulthood	↑ Aβ1–42 (late)	↑ p-Tau (Thr181)	↑ Microglial genes; minocycline-sensitive	Long-term vulnerability	Microglial priming (immune node)	Barnett et al., 2022
3xTg-AD	Chronic binge + abstinence	Midlife → aging	–	↑ Tau; ↑ AT8	↓ LAMP1; DAM signature	Sex-specific deficits	Lysosomal dysfunction (metabolic node)	Tucker et al., 2022
APP/PS1	Moderate chronic (2-bottle choice)	10 weeks	↑ Plaque number; ↓ size	–	Metabolic dysfunction	Behavioral changes	Aggregation dynamics	Day et al., 2023
APP/PS1	Repeated binge	1 month	↑ Plaque burden	–	↑ NLRP3, ASC, IL-1β	–	Inflammasome activation (immune node)	Brezani et al., 2025
Tg2576	Lieber–DeCarli diet (5%)	6 weeks	↑ Insoluble Aβ1–42	–	Liver injury correlation	–	Systemic–brain coupling	Chandrashekar et al., 2025
APP/PS1	Low–moderate ethanol	Presymptomatic	↓ Plaques; ↓ cytokines	–	↓ IL-1β, TNF-α	Improved cognition	Anti-inflammatory shift (dose effect)	Kang et al., 2024
Aged WT/AD models	Binge ethanol	Late-life	–	↑ p-Tau	↑ Microglial reactivity; NLRP3	Memory deficits	Age-dependent vulnerability	Anton et al., 2024
Rat (non-transgenic)	Liquid diet ethanol	∼5 weeks	↑ APP, BACE1	–	↑ Oxidative stress	–	APP processing (production node)	Kim et al., 2011
AD rat (TgF344-AD)	Chronic intermittent	Lifespan	Variable	Variable	Sex-dependent inflammation	Anxiety/cognitive shifts	Sex-specific modulation	Marsland et al., 2025
Mixed rodent models	Chronic ethanol	Variable	Variable	Variable	↑ Neuroinflammation	Impaired plasticity	Synaptic dysfunction	Mira et al., 2020
5xFAD	Binge ethanol	Adult	–	–	No major microglial change reported	Memory impairment	Regional plaque modulation (metabolic/aggregation)	Le et al., 2025

Aβ, amyloid-β; Aβ42/40, amyloid-β 42/40 ratio; AD, Alzheimer’s disease; AIE, adolescent intermittent ethanol exposure; Akt, protein kinase B; APP, amyloid precursor protein; ASC, apoptosis-associated speck-like protein containing a CARD; AT8, phosphorylated Tau epitope (Ser202/Thr205); BACE1, β-site APP-cleaving enzyme 1; CNS, central nervous system; DAM, disease-associated microglia; IL-1β, interleukin-1 beta; LAMP1, lysosome-associated membrane protein 1; mTOR, mechanistic target of rapamycin; NLRP3, NOD-, LRR-, and pyrin domain-containing protein 3; p-Tau, phosphorylated Tau; TNF-α, tumor necrosis factor alpha; WT, wild type; 3xTg-AD, triple transgenic Alzheimer’s disease mouse model (APP, PS1, Tau); APP/PS1, amyloid precursor protein/presenilin-1 transgenic mouse model; Tg2576, amyloid precursor protein Swedish mutation mouse model; TgF344-AD, Fischer 344 rat Alzheimer’s disease model; 5xFAD, five familial Alzheimer’s disease mutations mouse model.

Developmental exposure models further support a sustained neuroimmune component. Adolescent intermittent ethanol produces persistent increases in Aβ1–42 and p-Tau (Thr181) detected in adulthood, accompanied by upregulation of microglial and inflammatory transcripts; attenuation of these effects by minocycline, a brain-penetrant tetracycline antibiotic that inhibits microglial activation and neuroinflammatory signaling, is consistent with a causal contribution of prolonged neuroimmune activation (Barnett et al., 2022). While inflammatory signaling provides a mechanistic link between ethanol exposure and protein aggregation, it also helping to explain variability across experimental paradigms. Repeated binge exposure amplifies NOD-like receptor family pyrin domain containing 3 (NLRP3) inflammasome activation, apoptosis-associated speck-like protein containing a caspase recruitment domain (ASC) aggregation, and IL-1β release alongside increased amyloid pathology (Brezani et al., 2025). Ethanol also induces endoplasmic reticulum stress (ER), calcium dysregulation, and mitochondrial damage in glial cells, further promoting conditions that favor aggregation (Sushma et al., 2023). However, not all studies report uniform increases in Aβ pathology. Low-to-moderate ethanol exposure in presymptomatic APP/PS1 mice has been associated with reduced plaque burden and decreased Aβ1-40/42 levels, accompanied by dampened IKK–NF-κB signaling and lower IL-1β and TNF-α expression, particularly in males (Kang et al., 2024). These contrasting findings suggest that the effects of ethanol depend on exposure intensity, timing, and disease stage, with lower exposure conditions potentially engaging anti-inflammatory or clearance-supporting pathways, whereas higher or later exposures promote inflammasome activation and impaired clearance. This variability reflects a broader lack of consensus in the field, where differences in experimental design, including exposure paradigm, age at exposure, and sex, likely contribute to divergent outcomes. This pattern indicates that ethanol does not exert a uniform effect on Aβ pathology but instead modulates underlying neuroimmune and metabolic states that determine whether aggregation is promoted or attenuated, with similar context-dependent implications for Tau-related pathology through shared inflammatory and signaling mechanisms.

In addition to immune activation, impaired lysosomal functions, contribute to the increase in Aβ and Tau accumulation. Evidence from Aβ-focused transgenic models extends this framework by highlighting changes in plaque architecture and metabolic coupling. In APP/PS1 (amyloid precursor protein/presenilin-1) mice, Day et al. (2023) exposed 5.5-month-old animals to a 10-week modified two-bottle choice paradigm (20% w/v ethanol for 12 h/day during the dark cycle, four consecutive days per week) and observed an increase in Aβ plaque number, accompanied by a redistribution toward smaller plaque sizes. These neuropathological changes co-occurred with signals of brain atrophy and peripheral metabolic dysfunction, including glucose intolerance. Notably, canonical APP-processing enzymes, β-site amyloid precursor protein–cleaving enzyme 1 (BACE1) and a disintegrin and metalloproteinase domain-containing protein 10 (ADAM10), were not detectably altered, while insulin-degrading enzyme IDE showed a downward trend (Day et al., 2023). On the other hand, a study in non-transgenic rat model has reported increases in hippocampal BACE1 and APP expression following 5 weeks of an ethanol-containing liquid diet, with a concomitant increase in oxidative stress. In that context, ethanol was proposed to enhance oxidative stress-dependent regulation of BACE1 activity, supported by increased expression of proteins involved in γ-secretase complex assembly and BACE1-associated oxidative regulation, including PS1 and nicastrin (Kim et al., 2011). When considered alongside APP/PS1 studies in which plaque burden increased without detectable changes in BACE1 or ADAM10 expression, these findings indicate that ethanol does not exacerbate Aβ pathology through a single, uniform mechanism. In models lacking transgene-driven Aβ overproduction, ethanol can enhance BACE1 activity through oxidative stress-dependent pathways, thereby increasing Aβ generation. By contrast, in APP/PS1 mice, where amyloidogenic drive is already elevated, ethanol-associated increases in plaque burden appear to arise independently of further APP-processing enzyme upregulation, implicating impaired clearance, altered aggregation dynamics, or disrupted metabolic-immune coupling as dominant contributors.

Of note, chronic-binge exposure (5 g/kg/day, intragastric; 5 days on/2 days off from 5.5 to 9 months) followed by abstinence leads to enduring Tau elevations and disease-associated microglial (DAM) signatures, together with reduced lysosomal marker-associated membrane protein (LAMP)-1, indicating compromised lysosomal capacity and altered glial states (Tucker et al., 2022). These findings suggest that ethanol exposure induces a persistent neuroimmune–lysosomal dysfunction that may prime the brain for later protein accumulation rather than acting solely as an acute trigger, with potential downstream effects on both Aβ clearance and Tau accumulation through shared proteostatic pathways.

In addition to the processes discussed above, altered microglial phagocytic function contributes directly to Aβ accumulation. Microglia are the principal phagocytic cells of the CNS and maintain Aβ homeostasis by internalizing extracellular amyloid species and targeting them for lysosomal degradation through receptor-mediated mechanisms involving triggering receptor expressed on myeloid cells 2 (TREM2), and scavenger receptors such as SCARA1/MSR1, CD36, and SR-BI/SCARB1 (Njoku and Kanmogne, 2025). Experimental evidence shows that disruption of these pathways reduces Aβ internalization and exacerbates plaque accumulation, while some receptors simultaneously mediate pro-inflammatory signaling (Njoku and Kanmogne, 2025; Wilkinson and El Khoury, 2012). Ethanol exposure appears to alter these processes at multiple levels. In APP/PS1 models, ethanol perturbs receptor-mediated transport systems including low-density lipoprotein receptor-related protein-1 and the receptor for advanced glycation end products, which regulate Aβ exchange across the neurovascular unit (Boreland et al., 2025; Clugston et al., 2014; Vetreno et al., 2013). In parallel, ethanol suppresses phagocytic machinery and lysosomal competence while promoting inflammation as evident from diminished microglial uptake of oligomeric Aβ and increased TLR4-NF-κB signaling, (Kalinin et al., 2018). These functional deficits align with transcriptional profiles observed after adolescent intermittent ethanol exposure, where persistent inflammatory activation coincides with elevated neurotoxic Aβ species (Chandrashekar et al., 2023). This integrated disruption of receptor-mediated uptake, enzymatic degradation, and inflammatory signaling supports a model in which ethanol impairs Aβ clearance through coordinated effects on microglial and metabolic pathways, with secondary consequences for Tau homeostasis due to impaired proteostatic capacity.

### 2.4 Ethanol-induced tauopathy

Ethanol exposure has been consistently associated with increased Tau phosphorylation and cleavage, implicating disruption of kinase–phosphatase balance as a central mechanism. Across *in vivo* models and neuronal systems, ethanol enhances the activity of key Tau kinases, including glycogen synthase kinase-3β (GSK3β), c-Jun N-terminal kinase (JNK), and cyclin-dependent kinase 5 (CDK5), while reducing phosphatase activity, particularly protein phosphatase-2A, thereby shifting the equilibrium toward sustained Tau phosphorylation (Fang, 2017; Saito et al., 2016). These changes are observed alongside impairments in memory and synaptic function and are frequently accompanied by oxidative stress and lipid peroxidation, suggesting that redox imbalance contributes to kinase activation and phosphatase suppression. This shift in enzymatic balance provides a direct mechanism by which ethanol promotes sustained Tau pathology.

In addition to phosphorylation, Tau undergoes acetylation, a post-translational modification (PTM) that influences its stability, aggregation propensity, and degradation (Cohen et al., 2011; Cook et al., 2014; Min et al., 2010; Shi and Tu, 2015). Experimental evidence indicates that acetylated Tau species exhibit reduced turnover and impaired degradation, consistent with a stabilizing effect on pathological Tau. In an htau mouse model, acute ethanol exposure increased acetylated Tau (TauK174ac) and prolonged its half-life, supporting a role for acetylation in limiting Tau clearance (Sabir et al., 2025). Although ethanol-derived acetate appears to preferentially support histone acetylation rather than direct Tau modification in this context, these findings suggest that ethanol perturbs acetylation-dependent proteostasis. However, direct *in vivo* evidence linking ethanol or HFD exposure to Tau acetylation in humans remains unavailable, and current mechanistic interpretations are derived primarily from preclinical animal and cellular models.

Mechanistic studies further indicate that ethanol promotes calpain-mediated cleavage of the CDK5 regulatory subunit p35 into p25, resulting in prolonged kinase activity and persistent Tau phosphorylation (Camp et al., 2006; Kusakawa et al., 2000). In parallel, chronic ethanol exposure disrupts antioxidant defenses and enhances excitotoxic signaling, conditions that favor activation of stress-responsive kinases and reduced phosphatase function (Barnett et al., 2022; Tucker et al., 2022). These observations indicate that ethanol-induced Tau modification is sustained by coordinated kinase activation and impaired dephosphorylation rather than transient signaling effects.

Importantly, kinase-driven Tau dysregulation appears to intersect with the microglial and metabolic pathways described in section “2.3 Preclinical evidence from animal models.” Inflammatory signaling can potentiate kinase activation, while lysosomal impairment may limit the clearance of modified Tau species, reinforcing accumulation. Thus, ethanol-induced tauopathy is best understood as the result of a coordinated shift in kinase–phosphatase balance operating within a broader context of neuroimmune activation and proteostatic disruption. Although these mechanisms are supported by multiple experimental models, direct evidence linking them within a single integrated framework remains limited, underscoring the need for studies that simultaneously assess signaling, clearance, and protein aggregation outcomes. Taken together, available evidence indicates that Tau pathology is characterized by interacting signaling, immune, and metabolic disturbances, rather than a single dominant pathway, although the relative contribution of each component remains incompletely defined.

### 2.5 Cell-based evidence

Cell-based models have been instrumental in delineating how ethanol directly perturbs AD-relevant pathways, including amyloidogenic processing, Tau regulation, and inflammatory signaling. Interpretation of these systems, nonetheless, requires caution, as ethanol rapidly evaporates under standard culture conditions (37 °C) and is not subject to systemic metabolism, limiting their utility for modeling *in vivo* exposure dynamics. Their principal value, therefore, lies in mechanistic dissection rather than quantitative extrapolation.

Across neuron-like cell systems, multiple studies indicate that ethanol can bias APP processing toward the amyloidogenic pathway by increasing BACE1 expression and Aβ output (Gabr et al., 2017; Huang et al., 2018). In human SK-N-MC neuroblastoma cells, ethanol increased BACE1 in a dose-dependent manner and elevated Aβ production, accompanied by ROS induction, eIF2α phosphorylation, and C/EBP homologous protein expression, consistent with activation of the integrated stress response and ER stress signaling (Gabr et al., 2017). Pharmacological and genetic perturbations in this system supported a defined causal sequence in which ethanol-induced eIF2α phosphorylation increased cyclooxygenase-2 (COX-2) expression and prostaglandin-E2 production. Subsequent signaling through the EP2 receptor activated protein kinase and CREB, leading to the transcriptional upregulation of BACE1 (Gabr et al., 2017). This work therefore links cellular stress responses to lipid mediator signaling and BACE1 induction, rather than reporting a purely correlative association. Comparable effects have been observed in other neuron-like human cell systems engineered to express mutant APP and BACE1. In HEK293 embryonic kidney cells and SH-SY5Y neuroblastoma cells, ethanol exposure over a range of concentrations (up to 139 mM) increased APP and BACE1 expression and elevated Aβ42 and Aβ40 levels, indicating that ethanol promotes amyloidogenic processing *in vitro* across distinct cellular models (Huang et al., 2018), highlighting how ethanol engages intracellular stress and signaling pathways that regulate amyloidogenic processing and proteostatic balance.

**FIGURE 1:**
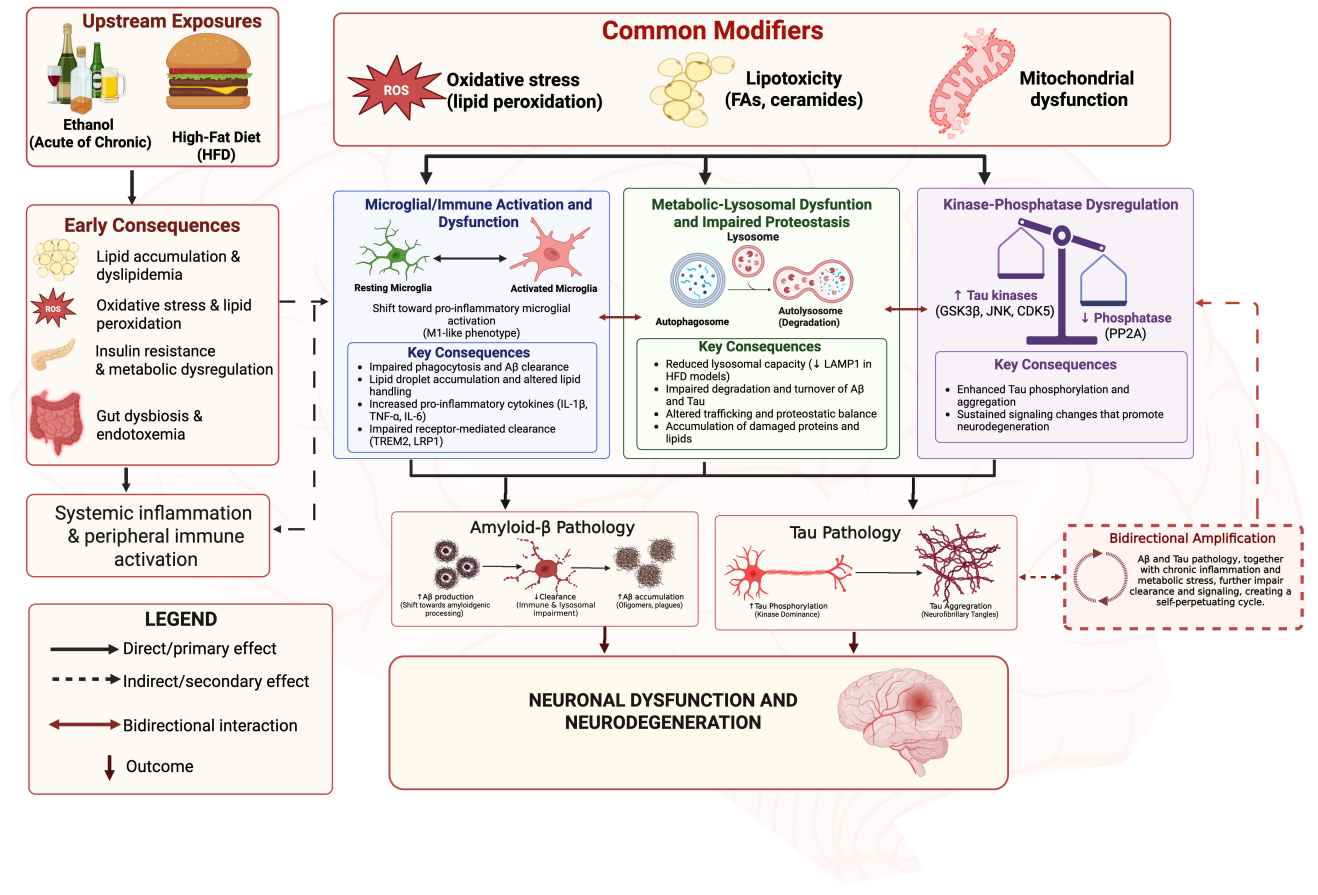
Convergent neuroimmune and metabolic mechanisms linking ethanol and high-fat diet to Alzheimer’s disease–related pathology. Schematic representation of how ethanol exposure and high-fat diet (HFD) induce metabolic and inflammatory stressors, including lipid accumulation, oxidative stress, insulin resistance, and systemic inflammation, that impact the central nervous system. These perturbations converge on interconnected pathways involving microglial activation, metabolic–lysosomal dysfunction, and kinase–phosphatase imbalance. Microglia shift toward a pro-inflammatory state with impaired amyloid-β (Aβ) clearance and increased cytokine production, while lysosomal dysfunction reduces degradative capacity and disrupts proteostasis, promoting accumulation of Aβ and Tau. Concurrently, dysregulation of Tau-related kinases and phosphatases favors sustained Tau phosphorylation. These processes interact to drive Aβ aggregation, Tau pathology, chronic neuroinflammation, and ultimately synaptic dysfunction and neurodegeneration. Created in BioRender.com.

Beyond neuronal cells, other brain cell types also contribute to APP processing and neuroinflammation. In human primary astrocytes, ethanol exposure increased APP and BACE1 expression and elevated Aβ1–42 levels, concomitant with robust induction of the pro-inflammatory cytokines TNF-α, IL-1β, and IL-6 and the lipid peroxidation marker 4-HNE. Notably, these responses were accompanied by upregulation of the long non-coding RNA BACE1-AS, implicating this transcript as a potential regulatory mediator of ethanol-induced amyloidogenic and inflammatory pathways (Kumar et al., 2024). Independent mechanistic studies demonstrate that ethanol increases astrocyte extracellular vesicle (EV) release and alters EV cargo composition, enriching these vesicles in TLR4, NFκB-p65, IL-1R, caspase-1, and NLRP3, while shifting EV-associated miRNAs such as miR-146a, miR-182, and miR-200b. These astrocyte-derived EVs are subsequently internalized by neurons, where they elevate neuronal COX-2 expression and miR-146a levels and reduce neuronal viability in a TLR4-dependent manner (Ibáñez et al., 2019). These findings indicate that ethanol-driven intercellular signaling amplifies inflammatory pathways and propagates dysfunction across cell types, linking immune activation with altered proteostasis and neuronal vulnerability. The effects of ethanol in promoting AD-like alterations in various *in vitro* and *ex vivo* models are summarized in Table 2. A schematic overview of ethanol-induced neuroimmune and metabolic mechanisms relevant to AD is presented in Figure 1.

**TABLE 2:** Cell-based studies examining ethanol-induced alterations in AD-relevant pathways.

Cell type/model	Ethanol (or acetaldehyde) concentration	Exposure duration	Dose-dependent	AD-relevant endpoints assessed	Principal findings	References
Human SK-N-MC neuroblastoma	Ethanol: 25–100 mM	24–72 h	Yes	BACE1, Aβ, ROS, eIF2α, CHOP, COX-2/PGE2	Ethanol increased BACE1 and Aβ production in a dose-dependent manner, accompanied by oxidative and ER stress markers	Gabr et al., 2017
SK-N-MC cells	Ethanol: 34, 69, 103 mM; acetaldehyde: 54–215 μM	24 h	Yes	BACE1, APP-C99 fragment	Dose-dependent increases in BACE1 and C99; effects attenuated by catalase inhibition	Huang et al., 2018
Primary rat microglia	Ethanol: 75 mM	24 h	No	Aβ1–42 phagocytosis	Ethanol reduced microglial uptake of oligomeric Aβ	Kalinin et al., 2018
Primary rat microglia (modular chambers)	Ethanol: 75 mM	24 h	No	Aβ phagocytosis	Confirmed impaired Aβ clearance under minimized ethanol evaporation	Kalinin et al., 2018
Primary astrocytes	Ethanol: 50–100 mM	24–48 h	Partial	EV release, inflammatory cargo, TLR4 signaling	Ethanol increased release of pro-inflammatory astrocyte EVs via TLR4	Ibáñez et al., 2019
Neuronal cultures (Tau-expressing)	Ethanol: 50–100 mM	24–48 h	Partial	p-Tau, caspase-3 cleavage	Ethanol-induced Tau phosphorylation and caspase-3–mediated Tau cleavage	Saito et al., 2010
Human Tau-inducible neuroblastoma (M1C)	Ethanol: 1.25–5 mg/mL (27–109 mM)	24–72 h	Yes	Total Tau, viability	Ethanol increased Tau accumulation without increasing Tau mRNA	Gendron et al., 2008
Organotypic neonatal rat hippocampal slices	Ethanol: 50–100 mM	Several days	No	p-Tau (Thr231)	Sustained Tau phosphorylation following ethanol exposure	Bailey et al., 2022
Mouse N2a-APP cells ± APOE4	Ethanol: 400 mM; APOE4: 7.5 μg/mL	24 h	No	ROS, apoptosis	Synergistic increase in oxidative stress and neuronal death with ethanol + APOE4	Li and Cheng, 2018
HEK293, PC12 cells	Ethanol: 10 mM	Pre-in cubation	No	Aβ aggregation, toxicity	Low-dose ethanol reduced Aβ oligomerization and toxicity	Ormeño et al., 2013
Primary rat hippocampal neurons	Ethanol: 10 mM	Pre-treatment	No	Synaptotoxicity	Low-dose ethanol attenuated Aβ-induced synaptic damage	Muñoz et al., 2015
Hippocampal–entorhinal slices	Ethanol: 20–30 mM	Pre-treatment	No	Neurotoxicity	Reduced Aβ-induced toxicity	Belmadani et al., 2004

Aβ, amyloid-β; Aβ1–42, amyloid-β peptide spanning residues 1–42; AD, Alzheimer’s disease; APOE4, apolipoprotein E ε4 isoform; APP, amyloid precursor protein; APP-C99, 99-amino-acid C-terminal fragment of amyloid precursor protein generated by β-secretase cleavage; BACE1, β-site amyloid precursor protein–cleaving enzyme 1; CHOP, C/EBP homologous protein; COX-2, cyclooxygenase-2; eIF2α, eukaryotic initiation factor 2 alpha; ER, endoplasmic reticulum; EVs, extracellular vesicles; HEK293, human embryonic kidney 293 cells; M1C, inducible human Tau-expressing neuroblastoma cell line; mRNA, messenger RNA; N2a-APP, mouse neuroblastoma Neuro-2a cells expressing mutant human amyloid precursor protein; p-Tau, phosphorylated Tau; ROS, reactive oxygen species; SK-N-MC, human neuroblastoma cell line; TLR4, toll-like receptor 4.

Collectively, ethanol exposure engages all three proposed mechanistic nodes, although the strength of evidence differs across domains. Direct evidence is strongest for microglial/immune activation, where multiple *in vivo* and *in vitro* studies demonstrate increased cytokine production, inflammasome signaling, and impaired phagocytosis. Substantial, though partly indirect, evidence supports disruption of metabolic–lysosomal function, including reduced lysosomal markers, impaired Aβ degradation, and altered proteostatic pathways. Evidence for kinase–phosphatase imbalance is supported by measured increases in Tau phosphorylation and kinase activation. In other cases, studies report alterations in related signaling pathways (Akt/mTOR, inflammatory signaling) without directly measuring kinase or phosphatase activity, limiting mechanistic resolution. Together, these findings suggest that ethanol perturbs interconnected immune, metabolic, and signaling systems that regulate Aβ and Tau homeostasis, with varying degrees of direct experimental support across nodes.

## 3 High-fat diet as a driver of AD-like pathology

Chronic consumption of a high-fat diet (HFD) leads to sustained metabolic stress states characterized by excess lipid accumulation, insulin resistance, dyslipidemia, and persistent low-grade systemic inflammation (You et al., 2025). These conditions are highly prevalent in aging populations and are increasingly recognized as modifiers of late-life cognitive trajectories. Epidemiological studies consistently identify midlife obesity, metabolic syndrome, and type-2 diabetes as risk factors for subsequent cognitive decline and dementia, suggesting that long-term metabolic dysregulation contributes to brain vulnerability well before the onset of clinical symptoms (Kouvari et al., 2022; Morys et al., 2021). Importantly, these associations persist after adjustment for traditional vascular risk factors, supporting a role for metabolic stress as a biologically relevant exposure rather than a simple correlate of comorbidity.

At the brain level, chronic HFD exposure disrupts homeostasis by promoting lipid accumulation in microglia, which impairs mitochondrial fatty acid β-oxidation and generates oxidative and metabolic stress. This dysfunction drives a pro-inflammatory microglial phenotype, increasing secretion of cytokines such as IL-1β, TNF-α, and IL-6, while simultaneously facilitating Tau hyperphosphorylation and AD-like pathology (Song et al., 2025; Figure 1). HFD also compromises cerebrovascular integrity, allowing peripheral inflammatory mediators and lipids to infiltrate the CNS, and impairs neuronal glucose metabolism, further reducing energy availability (Ge et al., 2025; Li F. et al., n.d.). Together, these interconnected disturbances create a feed-forward loop in which metabolic, oxidative, and inflammatory stress converge to exacerbate Tau pathology, synaptic dysfunction, and neuroinflammation, reflecting coordinated effects across metabolic–lysosomal, microglial/immune, and intracellular signaling pathways relevant to Aβ and/or Tau regulation. Neuroimaging and postmortem studies link metabolic syndrome to reduced hippocampal volume, white-matter abnormalities, and impaired cerebral glucose utilization, changes that overlap with early features of AD (Gómez-Apo et al., 2021; Zimmerman et al., 2021). However, direct human evidence linking dietary fat intake to Aβ or Tau pathology remains limited, as most clinical studies assess global cognition, or structural brain changes rather than AD-defining biomarkers. As a result, mechanistic insight into how HFD influences Aβ and/or Tau homeostasis has relied heavily on controlled experimental models.

### 3.1 Preclinical evidence from animal models

Across APP/Aβ-driven and Tau-relevant models, controlled HFD exposure has been used to directly test whether metabolic stress is sufficient to exacerbate AD-like pathology and cognitive dysfunction. Representative preclinical studies, including diet composition, exposure window, and major pathological and behavioral outcomes, are summarized in Table 3. In 3xTg-AD mice, Julien et al. (2010) showed that a HFD (35% w/w), in the context of a low n-3:n-6 polyunsaturated fatty acid (PUFA) ratio, aggravated both Aβ and Tau pathologies across a long exposure window (4–13 months of age). Another study by Knight et al. (2014) in 3xTg-AD showed a dissociation between cognition and classical pathology. They reported that HFD accelerated and intensified memory impairments in 3xTg-AD (and affected non-transgenic controls) without parallel changes in Aβ/Tau readouts. These findings point to neuroinflammation and metabolic dysfunction as proximal drivers of behavioral vulnerability (Knight et al., 2014). Barron et al. (2013) provide clear evidence that sex modifies the metabolic response to high fat diet exposure in 3xTg-AD mice. Both male and female animals developed increased body weight and showed worsening Alzheimer related outcomes, including impaired Y maze performance and elevated hippocampal Aβ. The metabolic phenotype, however, diverged substantially. Male mice displayed a pronounced metabolic syndrome–like profile, with elevated fasting glucose, increased insulin levels, and greater insulin resistance. Female mice, despite comparable weight gain, remained relatively protected from these glycemic and insulin disturbances under intact hormonal conditions. Findings from ovariectomized females indicate that this protection is at least partly mediated by ovarian hormones (Barron et al., 2013). Vandal et al. (2014) employed a “diabetes–AD” coupling strategy in 3xTg-AD mice, using HFD to induce metabolic dysfunction and subsequently testing whether insulin administration could reverse downstream effects. Their findings suggest that insulin signaling is not merely associated with AD-related pathology but can causally modulate brain Aβ-related endpoints and cognitive performance (Vandal et al., 2014). In App^*NL*–*F*/*NL*–*F*^ knock-in mice, Mazzei et al. (2021) demonstrated that a prolonged HFD induced obesity and type-2-diabetes-like metabolic impairments in both wild-type and App^*NL*–*F*/*NL*–*F*^ mice; however, only the App^*NL*–*F*/*NL*–*F*^ cohort exhibited cognitive impairment accompanied by pronounced increases in Aβ deposition, microgliosis, and hippocampal insulin resistance. These findings suggest that genotype and baseline pathological burden determine whether metabolic stress imposed by diet translates into plaque-relevant and cognitive outcomes (Mazzei et al., 2021). These findings collectively suggest that HFD acts as a context-dependent modifier of AD-like pathology, with effects mediated through metabolic stress, neuroimmune activation, and signaling pathways influencing Aβ and Tau homeostasis.

**TABLE 3:** Preclinical studies linking HFD to AD-like pathology.

Model	Diet composition	Exposure window	Aβ /plaques/CAA	Tau endpoints	Neuroinflammation/ glia	Cognitive/behavioral outcomes	References
3xTg-AD	HFD vs. normal diet	3 months	↑ Amyloid deposition	↑ Tau phosphorylation	↑ Microgliosis; lipid-droplet microglia; T-cell infiltration	Impaired spatial memory (MWM)	Liang et al., 2024
APP/PS1	HFD	Chronic feeding	↑ AD-related neuropathology	NA	Not reported	Deficits in memory, social, sensorimotor behavior	Bracko et al., 2020
APP/PSEN1	60% kcal fat	10 months	↑ Aβ burden; reversible with diet normalization	NA	↑ Inflammatory markers	Y-maze, nesting, locomotor deficits	Walker et al., 2017
3xTg-AD	35% w/w fat vs. 5% w/w, low n-3:n-6 PUFA	4–13 months	↑ Aβ pathology	↑ Tau pathology	NA	Memory impairment	Julien et al., 2010
3xTg-AD; WT	60% kcal fat vs. control	From 2 months	No plaque/oligomer change	No Tau change	Early microglial activation	Earlier, more severe memory deficits	Knight et al., 2014
THY-Tau22	60% kcal fat	Across 3–11 months	Not primary	Tau pathology altered (sex-dependent)	↑ Astrogliosis/microgliosis	Behavioral deficits	Kacířová et al., 2021
5xFAD	HFD	10 weeks	↑ CAA and cerebrovascular Aβ	NA	NA	Cognitive impairment	Lin et al., 2016
APPswe/PS1	Western diet	4 months	No consistent ↑ Aβ	NA	BBB dysfunction; ↑ MMP-9	Accelerated cognitive decline	Thériault et al., 2016
APP/PS1	60% kcal fat	Chronic	↑ Cortical soluble Aβ	NA	NA	Cognitive impairment	Guo et al., 2020
APP/PS1	HFD	Long-term	↑ Aβ accumulation	NA	NA	Cognitive impairment	Bao et al., 2022
AppNL-F/NL-F KI	40% kcal fat + 0.15% cholesterol	12 months	↑ Hippocampal Aβ	NA	↑ Microgliosis; insulin resistance	Impaired cognition	Mazzei et al., 2021
Mouse AD-related model	60% kcal fat	Early intervention	AD-like pathology	Tau assessed	Glial activation	Cognitive deficits	Amelianchik et al., 2021
APP/PS1; WT	D12492 (60 kcal% fat)	From 10 weeks	AD-like pathology	NA	NA	Behavioral impairment	Lee et al., 2018
E3FAD vs. E4FAD	Western diet (high SFA/sugar)	12 weeks	APOE4-dependent ↑ pathology	NA	↑ Inflammatory signaling	Behavioral deficits	Moser and Pike, 2017
3xTg-AD	HFD	Short-term	Not primary	NA	↑ Complement-mediated microglial activity	Memory impairment	Mackey-Alfonso et al., 2024
5xFAD	HFD-induced obesity	Chronic	↑ Plaque-associated pathology	NA	↑ Glial reactivity	Not reported	Choi et al., 2022

AD, Alzheimer’s disease; HFD, high-fat diet; WD, Western diet; kcal%, percentage of total caloric content derived from fat; w/w, weight-by-weight percentage; PUFA, polyunsaturated fatty acids; n-3 and n-6, omega-3 and omega-6 fatty acids; APP, amyloid precursor protein; PS1/PSEN1, presenilin-1; APP/PS1 or APP/PSEN1, transgenic mouse models expressing mutant human APP and presenilin-1; 3xTg-AD, triple-transgenic Alzheimer’s disease model expressing mutant APP, PS1, and MAPT (Tau); THY-Tau22, Tauopathy model expressing mutant human Tau under the Thy1 promoter; 5xFAD, amyloid model carrying five familial AD mutations; App^NL-F/NL-F KI, APP knock-in model carrying Swedish (NL) and Iberian (F) mutations expressed from the endogenous locus; KI, knock-in; E3FAD and E4FAD, amyloid mouse models expressing human APOE3 or APOE4; Aβ, amyloid-β; plaques, parenchymal Aβ aggregates; CAA, cerebral amyloid angiopathy; MWM, Morris water maze; NA, not available.

It should be pointed out that an HFD not only increases Aβ deposition but also alters the specific regulatory nodes within APP processing. Using APP transgenic mice, Maesako et al. (2012) showed that HFD exacerbates AD-like pathology, worsening memory impairment while increasing soluble Aβ oligomers and amyloid deposition, and although they did not report a significant increase in total BACE1 protein levels, the elevated Aβ burden is consistent with a shift toward enhanced amyloidogenic processing, implying increased β-site cleavage of APP rather than changes in BACE1 abundance. Other groups have focused on post-translational regulation of BACE1. Bao et al. (2022) using the APP/PS1 model, reported that long-term HFD aggravated Aβ accumulation and cognitive impairment in association with increased BACE1 phosphorylation and SUMOylation, suggesting that “BACE1 upregulation” often reflects enhanced enzymatic activity or altered processing rather than mere increases in protein abundance. Guo et al. (2020) found that APP/PS1 mice maintained on a 60% kcal fat diet for 6.5 months exhibited cognitive deficits, increased cortical soluble Aβ, and elevated APP expression, positioning HFD as a driver of AD-like phenotypes in genetically susceptible animals. These studies indicate that HFD engages trafficking, post-translational and transcriptional mechanisms that influence amyloidogenic processing and/or expression.

Bracko et al. (2020) similarly reported that HFD intensified AD-related behavioral abnormalities and neuropathology in APP/PS1 mice but showed that this worsening could not be explained by further reductions in cerebral blood flow. This distinction helps clarify that the pathological effects of HFD extend beyond vascular hypoperfusion (Bracko et al., 2020). In addition, Walker et al. (2017) showed in APP/PSEN1 mice that long-term HFD induced cognitive impairment and inflammatory marker expression and that dietary reversal improved metabolic and inflammatory markers while reducing Aβ-related outcomes. These results support the view that at least part of the HFD-induced phenotype remains reversible and diet-responsive (Walker et al., 2017).

Notably, evidence from HFD models indicates that chronic metabolic stress undermines microglial Aβ clearance. In APP^*NL*–*G*–*F*^ knock-in mice maintained on a prolonged HFD, microglial recruitment to plaques is reduced and uptake of Aβ is impaired relative to controls, coinciding with intracellular lipid droplet accumulation and elevated cholesterol esters in microglia, a metabolic phenotype that correlates with depressed expression of phagocytosis-related transcripts and impaired migration toward amyloid deposits (Yang et al., 2025). HFD is associated with lower expression of lipid-sensing and phagocytic-linked receptors such as TREM2 in plaque-associated microglia, suggesting that obesogenic stress disrupts receptor-mediated engagement with Aβ and biases microglia toward dysfunctional, lipid-laden states (Yang et al., 2025). Of note, impairments in lysosomal functions contribute to lipid accumulation and defects in proteostasis. However, the link between lysosomal dysfunction and Aβ accumulation upon HFD feeding remains unclear. A study using APP/PS1 transgenic mice implicates broader microglial and inflammatory gene network changes under HFD, including altered expression of TREM2, CD68, CX3CR1, and other phagocytosis-related transcripts, further supporting the notion that metabolic stress shifts microglial phenotypes away from efficient amyloid clearance and toward inflammatory activation (Reilly et al., 2020). Taken together, these findings indicate that HFD promotes amyloidogenesis and synaptic disruption via BACE1 regulation and microglial remodeling, even in the absence of overt plaque accumulation. These findings support a model in which HFD shifts microglial states toward lipid-laden, clearance-impaired phenotypes while promoting inflammatory activation, linking metabolic dysfunction to impaired Aβ handling and broader proteostatic imbalance.

### 3.2 HFD-induced tauopathy

Regarding the role of HFD in promoting tauopathy, another pathologic process leading to AD, Kacířová et al. (2021) used THY-Tau22 mice, which express human Tau with G272V and P301S mutations under the neuron-specific Thy1.2 promoter and progressively develop Tau hyperphosphorylation and neurofibrillary tangles. They found that HFD-induced obesity and peripheral insulin resistance were accompanied by with females showing impaired short term spatial memory, increased microgliosis and astrogliosis, and elevated Tau phosphorylation at T231, whereas males displayed peripheral metabolic alterations but only modest or limited changes in neuroinflammation and Tau phosphorylation (Kacířová et al., 2021). Tau-relevant signaling changes have also been reported under HFD/high-cholesterol (HC) diet conditions in models not engineered for tauopathy. In WT C57BL/6 mice fed a defined HF/HC diet (21% fat, 1.25% cholesterol) for 2 months, Bhat and Thirumangalakudi (2013) documented increased Tau phosphorylation together with impaired brain insulin/IGF signaling and reduced synaptic protein levels. These findings link diet induced metabolic stress to an imbalance in Tau regulating kinases and phosphatases, particularly within the insulin and insulin like growth factor 1 signaling pathway and glycogen synthase kinase 3 axis (IIS GSK3 pathway), providing a mechanistic basis for Tau hyperphosphorylation (Bhat and Thirumangalakudi, 2013). Interestingly, longer-term HFD exposure in aged WT mice has been associated with cognitive decline accompanied by Tau hyperphosphorylation and microglial activation, indicating that aging may amplify diet-induced vulnerability even in the absence of AD-related transgenic drivers (Liang et al., 2023). These studies suggest that dietary metabolic perturbations can exacerbate Tau-associated neuroinflammation and phosphorylation cascades.

High-fat diet has been shown to simultaneously exacerbate amyloidosis and tauopathy, contributing to CNS dysfunction. In 3xTg-AD mice, Liang et al. (2024) reported that prolonged HFD consumption aggravated Aβ deposition, Tau phosphorylation, neuroinflammation, and cognitive deficits. These effects were linked to lipid-droplet-accumulating microglia and altered fatty-acid metabolism, one of the HFD-specific mechanisms leading to AD-like pathology (Liang et al., 2024). Notably, HFD-induced neural dysfunction can also manifest in the absence of measurable changes in amyloid or Tau. Knight et al. (2014) reported that in 3xTg-AD mice, HFD produced robust and lasting memory impairment associated with neuroinflammation but not necessarily accompanied by detectable changes in Aβ or Tau phosphorylation at the assayed timepoints. These findings suggest that HFD-driven cognitive impairment can occur through inflammatory and synaptic pathways that operate partly independently of plaque and tangle pathology, depending on the duration of dietary exposure and the timing of outcome assessment (Knight et al., 2014).

### 3.3 Cell-based evidence for HFD-induced dysregulation of AD pathways

Rather than modeling HFD exposure directly, cell-based studies have modeled metabolic stress by applying defined free fatty acids (FFA) to neuronal and glial systems, allowing dissection of lipid-specific effects on amyloidogenic processing, Tau regulation, and innate immune signaling relevant to AD. In foundational work, primary rat cortical astroglia were treated with palmitic acid (PA) delivered as a 0.2 mM bovine serum albumin-conjugated preparation for 24 h, and this significantly increased astrocytic *de novo* ceramide synthesis measured by high-performance liquid chromatography. Conditioned medium from these PA-activated astrocytes induced upregulation of BACE1, increased generation of Aβ (Aβ40 and Aβ42), and hyperphosphorylation of Tau at AD-relevant sites in primary cortical neurons, establishing a causal link between astroglial saturated fatty acid (SFAs) metabolism and prototypical AD molecular signatures in neurons (Patil et al., 2007). Interventions that inhibited astroglial ceramide synthesis blocked these effects, highlighting a mechanistic role for lipid metabolic intermediates in mediating proteostatic disturbance initiated within non-neuronal cells, rather than through direct cytotoxic effects at the neuronal membrane (Patil et al., 2007).

Consistent with this mechanism, Patil and Chan (2005) showed that direct exposure of neurons to PA or stearic acid did not increase Tau phosphorylation, whereas conditioned medium from FFA-treated astrocytes induced robust neuronal Tau hyperphosphorylation; co-treatment with the antioxidant N-acetyl cysteine attenuated this effect, implicating glia-mediated oxidative stress in Tau kinase activation. Astrocytes are metabolically equipped to take up and process FFAs at concentrations that neurons poorly tolerate, and in doing so, FFA exposure triggers mitochondrial β-oxidation overload, ER stress, and NADPH oxidase activity, producing ROS and lipid peroxidation products. These oxidative molecules act as paracrine signals to neurons, activating Tau-relevant kinases, whereas direct neuronal exposure fails to promote similar effects. Also, astroglial ceramide accumulation triggered by PA correlated with downregulation of glucose transporter 1 (GLUT1) expression and impaired glucose and lactate metabolism, findings that recapitulate aspects of metabolic dysfunction observed in AD pathology (Patil et al., 2007).

In a similar study, Kim et al. (2023) showed that palmitate combined with elevated glucose induces insulin resistance in primary rat cortical neurons and human cortical stem cells, as evidenced by increased p-IRS-1 and p-JNK. The increase in insulin resistance was associated with enhanced APP phosphorylation and altered APP processing, while also increasing APP secretion in EVs. When these EVs were applied to naïve neurons, they triggered Tau phosphorylation, linking metabolic stress to canonical AD proteinopathy pathways and highlighting insulin signaling deficit as a mechanistic bridge linking lipid overload with amyloidogenic APP handling and Tau dysregulation (Kim et al., 2023). In cell-free assays, Wilson and Binder showed that long-chain FFAs directly promoted Tau and Aβ aggregation. They showed that arachidonic acid (AA; ω-6 PUFA) induced Tau assembly at 10–20 μM, while oleic acid (OA; MUFA) and linoleic acid (LA; ω-6 PUFA) facilitated Aβ fibrillogenesis at 40 μM, as measured by thioflavin fluorescence and further confirmed by electron microscopy. These findings indicate that specific unsaturated FFAs can directly enhance the intrinsic polymerization of AD proteins, often more effectively than saturated fatty acids (Wilson and Binder, 1997). However, how different fatty acids alter these processes *in vivo* is still unclear.

Beyond neuronal responses, cellular microglial models indicate that SFAs directly modulate innate immune phenotypes relevant to AD-related neuroinflammation. Exposure of microglial cell lines (BV-2) and primary microglia to PA (100–300 μM) activates TLR4–NF-κB signaling, thereby increasing the pro-inflammatory cytokines such as TNF-α, IL-1β, and IL-6, along with nitric oxide and ROS production. These inflammatory changes can propagate neuronal dysfunction and have been mechanistically linked to enhanced Tau phosphorylation and plaque-associated inflammation in AD models. While these experiments do not directly measure Aβ or Tau, they illustrate how SFAs polarize microglia toward maladaptive immune states that may exacerbate proteostatic stress within neuronal networks (Wang et al., 2012). A similar effect was observed in HMC3 microglial cells, where PA exposure increased markers of inflammation and oxidative stress, including IL-6, MCP-1, COX-2, and lipid peroxidation, indicating that SFAs are potent inducers of glial inflammatory response (Chmielarz et al., 2023). In parallel, studies using MG6 microglial cells treated with oleic acid show that metabolic stress promotes lipid droplet accumulation and transcriptional reprogramming that impairs the cells’ capacity to phagocytose Aβ peptides. These effects coincide with the downregulation of genes involved in cholesterol efflux and phagocytic engulfment, suggesting that chronic lipid exposure undermines microglial homeostatic functions that normally constrain amyloid accumulation (Yang et al., 2025). These studies suggest that SFAs can directly modulate glial inflammatory response. However, some microglial models show that palmitate can enhance phagocytic uptake under specific conditions, highlighting a complex and context-dependent modulation of immune activation by lipid stress (Tracy et al., 2013).

Proximal to immune signaling, interventions with unsaturated fatty acids such as docosahexaenoic acid (DHA) demonstrate that lipid species themselves can modulate inflammatory and metabolic stress responses *in vitro*. In neuron-like and microglia-like cell lines, DHA pretreatment attenuates palmitate-induced pro-inflammatory cytokine expression, ER stress markers, and mitochondrial dysfunction, highlighting that cellular effects of fatty acids are lipid class specific. These observations, summarized in Table 4, align with broader literature showing that SFAs promote pro-inflammatory microglial polarization, whereas ω-3 PUFAs favor anti-inflammatory states (Butler et al., 2023; Sanjay et al., 2022). Although *in vitro* models isolate HFD effects from systemic endocrine, microbiome, and neurovascular interactions present *in vivo*, the evidence indicates that fatty acid induced metabolic stress promotes AD. related pathology through activation of intracellular stress kinases such as JNK, disruption of insulin signaling pathways, increased amyloidogenic processing via upregulation of BACE1 and altered APP handling, glia mediated induction of neuronal Tau hyperphosphorylation through oxidative stress and ceramide dependent mechanisms, and enhanced pro inflammatory responses in microglia via TLR4 NF κB signaling.

**TABLE 4:** Fatty acid–based *in vitro* models relevant to AD pathways.

Fatty acid (class)	Concentration and delivery	Exposure duration	Cell or assay system	AD-relevant readouts	Key findings	References
Palmitic acid (SFA)	0.2 mM, BSA-conjugated in culture medium	24 h	Primary rat astrocytes → conditioned medium applied to neurons	Ceramide synthesis, BACE1 levels, Aβ40/42, Tau p-epitopes	Astroglial PA increases ceramide; astrocyte-derived factors elevate neuronal BACE1 and Aβ, and drive Tau hyperphosphorylation via oxidative signaling	Patil et al., 2007
Palmitic acid (SFA)	100–300 μM, BSA-conjugated	24 h	Primary rat astrocytes; conditioned media to neurons	Tau phosphorylation (AT8, PHF-1), astroglial oxidative stress	FFA-treated astrocytes produce factors that induce Tau hyperphosphorylation in neurons; antioxidants attenuate effect.	Patil and Chan, 2005
Palmitic acid (SFA)	100 μM	24 h	SH-SY5Y-APP Swe neuroblastoma	BACE1 mRNA/protein, BACE1 activity, intracellular and secreted Aβ1–42	PA induces BACE1 expression/activity and increases amyloidogenic processing via ER stress pathways	Marwarha et al., 2017
Stearic acid (SFA)	100–300 μM, BSA-conjugated	24 h	Primary astrocytes; conditioned media to neurons	Tau p-epitopes, oxidative markers	Like PA, stearic acid triggers astrocytic stress that indirectly induces neuronal Tau hyperphosphorylation	Patil and Chan, 2005
Oleic acid (MUFA)	40 μM	Acute (minutes–hours)	Cell-free Aβ assembly assay	Thioflavin fluorescence; fibril morphology	OA promotes β-amyloid fibrillization similar to other unsaturated fats	Wilson and Binder, 1997
Linoleic acid (ω-6 PUFA)	40 μM	Acute	Cell-free Aβ assembly assay	Aβ fibrillization kinetics	LA, an abundant ω-6 PUFA, enhances Aβ polymerization *in vitro*	Wilson and Binder, 1997
Arachidonic acid (ω-6 PUFA)	10–20 μM	Acute	Cell-free Tau assembly assay	Thioflavin binding; filament formation	AA stimulates Tau filament assembly *in vitro* and can enhance polymerization of both Tau and Aβ	Wilson and Binder, 1997
Palmitic acid (SFA)	100–300 μM	12–24 h	Microglial cultures (BV2 / primary microglia)	IL-1β, TNF-α, NF-κB signaling (TLR4), nitric oxide, ROS	Saturated FAs activate microglial innate immune pathways that overlap with pro-inflammatory phenotypes observed in AD	Desale and Chinnathambi, 2020
Linoleic acid (ω-6 PUFA) and arachidonic acid (ω-6 PUFA)	Contextual comparisons in microglia	Variable	Microglial polarization frameworks	Cytokines, NF-κB, Tau p-kinases	ω-6 FAs tend to promote inflammatory signaling and are associated with Tau phosphorylation and aggregation pathways in microglial contexts	Desale and Chinnathambi, 2020
Palmitic vs. unsaturated FAs (comparative)	100 μM	24 h	SH-SY5Y types (APP, wild-type)	BACE1, APP processing shifts	SFAs drive amyloidogenic processing; unsaturated counterparts (oleate, palmitoleate) do not show same induction	Marwarha et al., 2017

HFD, high-fat diet; SFA, saturated fatty acid; MUFA, monounsaturated fatty acid; PUFA, polyunsaturated fatty acid; FFA, free fatty acid; APP, amyloid precursor protein; Aβ, amyloid-β peptide; Aβ1–42, amyloid-β peptide spanning residues 1–42; p-Tau, phosphorylated Tau; Tau p-epitopes, Tau phosphorylation at AD-relevant residues (AT8: Ser202/Thr205; PHF-1: Ser396/404); BACE1, β-site amyloid precursor protein–cleaving enzyme 1; ROS, reactive oxygen species; ER stress, endoplasmic reticulum stress; TLR4, toll-like receptor 4; NF-κB, nuclear factor kappa-light-chain-enhancer of activated B cells; IL-1β, interleukin-1 beta; TNF-α, tumor necrosis factor-alpha; BV2, murine microglial cell line.

## 4 Combined ethanol and HFD exposure

Evidence directly examining the combined effects of ethanol and HFD exposure on AD-related pathology remains limited, and most available studies focus on behavioral, inflammatory, or metabolic outcomes rather than canonical Aβ and Tau endpoints. Within this constraint, existing preclinical studies indicate that combined exposure is associated with exacerbation of CNS dysfunction, particularly in behavioral and neuroinflammatory domains. Singh et al. (2022) reported that chronic binge-like ethanol consumption markedly exacerbated the neurobehavioral and neurochemical abnormalities induced by long-term HFD in mice, including heightened oxidative stress, increased inflammation, pronounced microgliosis and astrogliosis, impaired neurogenesis, and disruptions in cholinergic signaling. Similarly, Gelineau et al. (2017) found that in C57BL/6J mice, combined exposure to HFD and ethanol produced distinct behavioral and physiological alterations, including impaired glucose tolerance, heightened anxiety-related behaviors, and shifts in neurotrophin and insulin signaling, that differed from the effects of either insult alone and exhibited notable sex-specific variability. Current evidence supports co-occurring and, in some cases, additive effects of ethanol and HFD exposure on neuroinflammation and metabolic dysfunction (Table 5). However, formal evidence for synergistic interactions remains limited, as most studies do not incorporate experimental designs that allow interaction effects to be quantitatively assessed.

**TABLE 5:** Preclinical studies examining combined ethanol and high-fat diet exposure and associated neurobiological outcomes.

Model	Exposure paradigm	Aβ outcomes	Tau outcomes	Neuroinflammation/ glia	Metabolic outcomes	Key findings	References
C57BL/6 mice	Chronic HFD + binge ethanol	Not directly measured	Not directly measured	↑ IL-1β, TNF-α, microgliosis, astrogliosis	↑ Oxidative stress; impaired antioxidant defenses; disrupted insulin signaling	Combined exposure exacerbates neuroinflammation and behavioral deficits	Singh et al., 2022
C57BL/6 mice	HFD + ethanol (chronic)	Not assessed	Not assessed	Not directly measured	Impaired glucose tolerance; altered insulin/neurotrophin signaling	Distinct metabolic and behavioral effects; sex-specific responses	Gelineau et al., 2017
C57BL/6 mice	Intragastric ethanol + HFD (hybrid model)	Not measured	Not measured	↑ P2X7, IL-1β, IL-6, MCP-1; neuronal loss	Not primary focus	P2X7-mediated neuroinflammation under combined exposure	Asatryan et al., 2015
BV2	Ethanol + ATP (P2X7 activation)	Not measured	Not measured	↑ IL-1β via P2X7	Not applicable	Ethanol enhances purinergic inflammatory signaling	Freire et al., 2019

Aβ, amyloid-β; AD, Alzheimer’s disease; HFD, high-fat diet; IL-1β, interleukin-1 beta; IL-6, interleukin-6; TNF-α, tumor necrosis factor alpha; P2X7, purinergic receptor P2X7; MCP-1, monocyte chemoattractant protein-1; ATP, adenosine triphosphate; ROS, reactive oxygen species; BV2, murine microglial cell line.

Mechanistically, these combined insults converge on shared inflammatory and metabolic pathways. Singh et al. (2022) reported increased NF-κB-activation and elevated IL-1β, TNF-α levels in the hippocampus, alongside, impaired antioxidant defenses. Concurrently, insulin/AKT signaling was disrupted, with reduced AKT activity which in turn, can increase GSK-3β activation, a pathway known to regulate Tau phosphorylation. Parallel dysregulation of Aβ metabolism was also observed: increased BACE1 activity and reduced markers of microglial clearance were reported, suggesting altered Aβ handling. These data therefore support co-occurring disruptions in Aβ- and Tau-relevant pathways, but do not yet establish causal or synergistic effects on protein aggregation. Gelineau et al. (2017) supported this model, showing that ethanol-HFD mice exhibited peripheral insulin resistance and altered central metabolic signaling, potentially amplifying neurodegenerative risk.

A particularly compelling line of evidence comes from Asatryan et al. (2015), who identified the purinergic P2X7 receptor as a critical node linking HFD and ethanol to amplified neuroinflammation. In their “Hybrid” model, combining chronic intragastric ethanol with HFD, purinergic receptor (P2X7P2X7) expression was strongly upregulated in hippocampal and striatal microglia (Asatryan et al., 2015). This upregulation correlated with elevated IL-1β, IL-6, MCP-1, iNOS, and pronounced neuronal loss, indicating a direct link between P2X7 activation and ethanol-diet-driven neurotoxicity. Pharmacological blockade of P2X7 significantly reduced inflammatory markers, demonstrating its functional role (Freire et al., 2019). Complementary *in vitro* studies show that ethanol potentiates ATP-induced P2X7-mediated pore formation and IL-1β release in microglia, indicating that ethanol can sensitizes cells to purinergic signaling. Although direct interactions between P2X7 and Aβ or Tau proteins were not measured in these studies, P2X7 activation is known to promote NLRP3 inflammasome activity, which has been independently linked to Aβ accumulation and Tau phosphorylation in other models. This positions P2X7 as a potential convergence point linking immune activation with proteinopathy-related pathways. Separate experimental studies have also implicated P2X7 signaling in ethanol-induced BBB dysfunction (Togre et al., 2024), although BBB integrity was not directly assessed in the combined HFD and ethanol paradigms discussed here. Despite these advances, the direct impact of combined HFD and ethanol exposure on canonical AD-related pathways, including Tau phosphorylation, Aβ processing, and GSK-3β-mediated signaling, remains insufficiently characterized, representing an important gap in mechanistic understanding.

Although ethanol and HFD exposure alone is known to impair microglial phagocytic capacity, no study to date has directly quantified how a combination of ethanol and HFD influences the expression, trafficking, or the function of key microglial scavenger receptors such as SCARA1/MSR1, CD36, or SR-BI *in vivo*. Moreover, no work has established a causal link between alterations in these receptors and deficits in Aβ clearance within AD models exposed to dual metabolic and ethanol stressors. This gap stands in contrast to the more extensive literature on TLR-dependent signaling, inflammasome activation, and global phagocytic impairments, and it underscores a critical unresolved question in understanding how lifestyle-associated risk factors converge on microglial proteostatic regulation.

**FIGURE 2:**
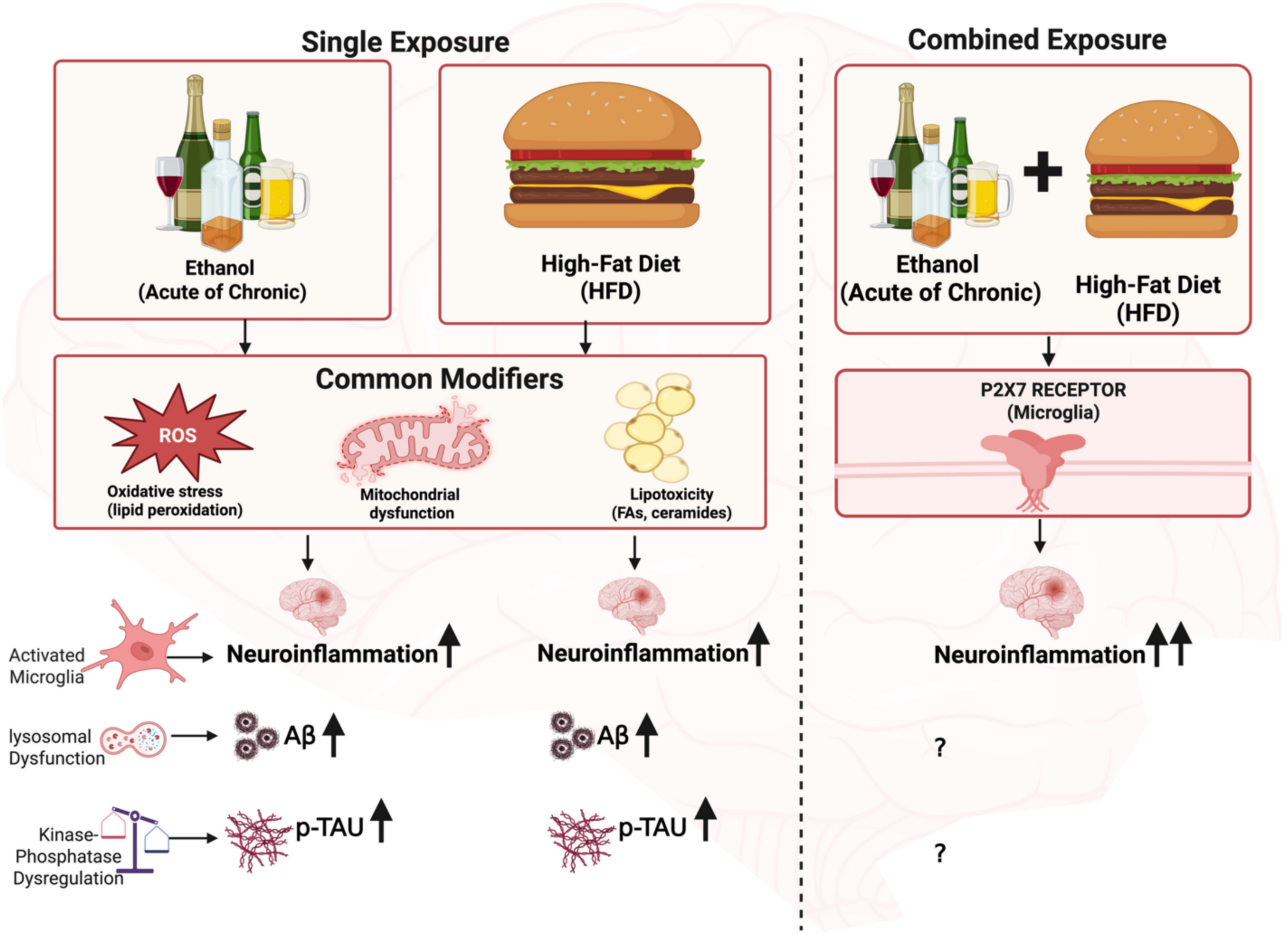
Proposed mechanisms by which ethanol and high-fat diet exposure contribute to Alzheimer’s disease-related neuropathology. The schematic summarizes the overlapping and potentially synergistic effects of ethanol exposure and high-fat diet (HFD) on neuroinflammatory and neurodegenerative pathways. Under single-exposure conditions, both ethanol (acute or chronic) and HFD induce common pathological modifiers, including oxidative stress/reactive oxygen species (ROS) generation, mitochondrial dysfunction, and lipotoxicity mediated by fatty acids (FAs) and ceramides. These alterations promote microglial activation and neuroinflammation, impair lysosomal function leading to increased amyloid-β (Aβ) accumulation, and disrupt kinase/phosphatase signaling pathways that favor Tau hyperphosphorylation (p-Tau). Under combined exposure conditions, ethanol and HFD may act synergistically to exacerbate neuroinflammation, potentially through activation of the microglial P2X7 receptor signaling axis. The downstream effects of combined exposure on Aβ accumulation and Tau pathology remain incompletely understood. Created in Biorender.com.

Despite these advances, direct evidence linking combined ethanol and HFD exposure to Aβ deposition, Tau phosphorylation, or Tau acetylation remains sparse. Few studies simultaneously assess protein aggregation, signaling pathways, and clearance mechanisms within a single experimental framework. This limits the ability to determine whether observed effects reflect true mechanistic convergence or parallel, independent processes. A schematic summary of the proposed intersections between ethanol and HFD exposure is presented in Figure 2.

## 5 Therapeutic implications, future directions, and conclusion

Accumulating preclinical evidence from largely single-exposure models indicates that ethanol and HFD influence overlapping microglial and metabolic pathways that impair amyloid and Tau homeostasis, rather than acting through isolated mechanism. Across rodent and cell-based models, both exposure engage innate immune signaling programs dominated by TLR4-NF-κB activation while suppressing transcriptional networks required for effective phagocytosis, lipid handling, and lysosomal degradation, including those associated with TREM2, CD36, and CD68 expression (Vetreno et al., 2013; Wilkinson and El Khoury, 2012). These changes coincide with reduced Aβ uptake, impaired lysosomal function, and sustained extracellular amyloid burden, alongside parallel effects on Tau phosphorylation pathways mediated by inflammatory kinase activation and disrupted phosphatase signaling. Notably, several studies demonstrate that metabolic- or ethanol-induced microglial reprogramming persists even after exposures cease, suggesting that these factors may lower the threshold for AD-like pathology by establishing a chronically pro-inflammatory, clearance-deficient microglial state (Barnett et al., 2022; Vetreno et al., 2013). However, direct evidence demonstrating synergistic effects under combined exposure conditions remains limited.

Therapeutically, these findings imply that targeting Aβ or Tau alone may be insufficient in metabolically compromised contexts unless paired with interventions that restore microglial immune-metabolic balance. Several candidate strategies have emerged. P2X7 receptor antagonists have been shown in preclinical models to reduce inflammasome activation and IL-1β release, suggesting potential to limit neuroinflammatory amplification, although clinical evidence remains limited (Alves et al., 2023; Freire et al., 2019). TREM2 agonists are being explored to enhance microglial phagocytosis and lipid handling, with emerging preclinical and early clinical data supporting their role in improving Aβ clearance (Nada et al., 2026; Yuan et al., 2025). PPARγ agonists, which regulate microglial lipid metabolism and promote lipid homeostasis, have demonstrated anti-inflammatory and metabolic benefits in preclinical neuroinflammatory models, with some studies also reporting reductions in pathological lipid-droplet accumulation; however, translation to human studies has produced mixed outcomes (Luo et al., 2025). Interventions targeting the gut–brain axis, including butyrate supplementation or probiotics, may reduce systemic inflammation and improve metabolic signaling (Li X. et al., n.d.), although evidence remains largely preclinical. In parallel, modulation of PTMs of Aβ and Tau represents an additional therapeutic avenue. While phosphorylation has been the primary focus, other PTMs; including acetylation, ubiquitination, and O-GlcNAcylation are increasingly recognized as critical regulators of protein aggregation and clearance (Alhadidy et al., 2025; Zafar et al., 2024). Site-specific inhibition of Tau kinases (GSK3β or CDK5) or enhancement of phosphatase activity may reduce pathological phosphorylation, although achieving specificity remains a challenge (Alquezar et al., 2021). Tau acetylation, which is influenced by acetyl-CoA availability and has been shown to impair protein degradation, represents a potential target for therapeutic modulation, particularly in the context of ethanol metabolism (Sabir et al., 2025).

Current evidence suggests that ethanol and HFD individually leads to (i) microglial/immune activation, (ii) metabolic–lysosomal dysfunction, and (iii) kinase–phosphatase imbalance which are associated with Aβ accumulation and tauopathy. Studies using a combination of HFD and ethanol showed enhanced neuroinflammation and behavioral abnormalities compared to individual exposure. However, the effect of combined exposure on the accumulation of Aβ and pathological tau remains to be studied. Within the proposed framework, available evidence supports contributions from all three mechanistic nodes; microglial/immune activation, metabolic-lysosomal dysfunction, and kinase-phosphatase imbalance; although support is strongest for inflammatory and metabolic pathways, with more limited direct evidence for Aβ and/or tau accumulation under combined exposure conditions. Advancing translational relevance will require integrated experimental and clinical approaches that simultaneously assess neuroinflammation, Aβ accumulation, and tau phosphorylation within the same framework. Key priorities include defining how specific lipid species and ethanol exposure patterns influence microglial state transitions, determining whether coordinated changes in Aβ and Tau PTMs occur under combined conditions, and establishing the reversibility of these processes. Incorporation of human-relevant biomarkers and longitudinal study designs will be essential for bridging preclinical findings with clinical populations and for developing mechanistically grounded strategies for prevention and intervention.
